# Editorial: Robotic evolution in genitourinary surgery: from a promising past to an even more exciting future

**DOI:** 10.3389/fsurg.2026.1977853

**Published:** 2026-09-10

**Authors:** Panagiotis Mourmouris, Nikolaos Kostakopoulos, Omer Argun

**Affiliations:** 1 Metropolitan Hospital, Neo Faliro, Greece; 2 Metropolitan General Hospital, Athens, Greece; 3 Istanbul kent University, İstanbul, Türkiye

**Keywords:** innovations, robotic, robotic partial nephrectomy, robotic radical prostatectomy, urology

Robotic surgery has undergone a tremendous transformation over the last ten years. What initially started as an extension of laparoscopy has evolved into a standalone platform capable of addressing complex surgical procedures. Even though this evolution seems to have reached its limits, new technologies and the use of AI promise an even more exciting future. The research topic “Robotic Evolution in Genitourinary Surgery: From Technical Innovation to Surgical Intelligence” was conceived to scientifically explore this expanding field of technology and to propose exciting novel ideas. The included contributions provide valuable insights on nearly all aspects of urological surgery along with surgical education and postoperative management of patients.

## Beyond minimal invasive surgery

The adoption of robotic platform was mainly driven by its ability to overcome the inherited limitations of conventional laparoscopy. Articulated instruments, elimination of hand tremors both enhanced with 3D vision improved ergonomics and introduced robot assisted laparoscopy mainly to prostate and renal surgery. The foundations for the next implosion have been placed: nowadays the question is no longer whether robotic surgeons can perform an even complex procedure, but whether they can invent better surgical strategies. The contemporary robotic surgeon is nowadays able to modify established surgical procedures based on patient's specific factors. Retzius sparing approach in robotic radical prostatectomy is a basic example. In their study, Zeng et al., compared Retzius- sparing and standard robotic radical prostatectomy in 120 patients. They concluded that the modified approach resulted in better early continence rates, maintaining the oncological and perioperative parameters of the procedure. We are aware of the advantages that this outcome can produce: Patients will choose a method that diminishes post-operative troublesome symptoms and enables their early return to their everyday life (Zeng et al.). The study reinforces one of the basic evolutionary steps of robotic surgery: enhancement of functional maintaining the oncological and surgical outcomes of a surgical procedure.

## Expanding boundaries

The most striking manifestation of robotic evolution is the progressive expansion of indications towards more complex and demanding procedures. Doganca et al. examined the role of PADUS and RENAL nephrometry scores in patients with large renal tumors. Authors concluded that robotic partial nephrectomy can be efficient and safe for tumors more complex than a simple small and exophytic renal mass. They also highlight one possible limitation of conventional anatomical scoring: complexity of renal masses could be underestimated without taking account of multifactorial determinants of the robotic approach (Doganca et al.). The abovementioned conclusion could open a debate about a possible transitions from static anatomical scores towards multidimensional and personalized risk models incorporating, except from the anatomy, more factors like patient characteristics, surgeons experience, imaging derived features and intraoperative parameters.

Perhaps an even more compelling example of the expanding boundaries of robotic surgery is provided by Gu et al. and Jia et al. who reported the use of the robotic platform for complex procedures like intracorporeal bilateral ileal ureter replacement for highly complex post radiation stricture complicated by uretero-arterial fistula and ileocystoplasty for bladder augmentation. As one can understand, these procedures are two of the most complex procedures in urological practice that require sophisticated reconstructive steps, involving precise dissection, suturing and tissue handling, demonstrating the significant improvements robotic technology has undergone.

## Precision requires navigation

The next generation of robotic surgery will increasingly depend on the intraoperative dynamic change of the anatomy rather than relying on surgeon's visual interpretation of solid postoperative images. The work of Sun et al. exploring binocular vision guidance in the female pelvis represent an example of the emerging direction. The development of more stable and precise visual navigation systems will surely facilitate accurate localization and manipulation within complex environments that will ultimately result in enhanced postoperative outcomes. Although such technologies remain at an early stage, their implications are substantial. The future operating room is unlikely to consist simply of a surgeon controlling robotic instruments while viewing a conventional endoscopic image. Instead, surgical navigation may increasingly integrate three-dimensional anatomical reconstruction, real-time image interpretation, augmented visualization, and intraoperative guidance. In this context, the robotic platform should be regarded not merely as a mechanical interface but as a potential surgical information platform.

## Personalization extends beyond operations

One of the most important goals of robotic platform is to improve the postoperative outcomes of a procedure and of course the quality of life of the patient after a major operation. The systematic review by Mourmouris et al. provides insights into one of the major factors that decreases quality of life of patients after radical prostatectomy: postoperative indwelling catheterization time. The study of more than 4,000 patients concluded that catheter removal on 3rd or 4th postoperative day (much earlier than the open procedure) after robotic radical prostatectomy is not only safe but also seems to enhance anastomosis recovery in selected patients (Sun et al.). This work illustrates an important point: improved surgical precision achieved by contemporary robotic approach helps surgeon's reconsider established peri and postoperative knowledge, based on individualized patient's characteristics and intraoperative factors.

## Robot and surgeon evolve together

Even though robots are evolving, the role of surgeon remains central and his training remains vital. More sophisticated cockpits in a plane demand a better trained pilot. The traditional training model, based predominantly on observation followed by progressively independent operating, is increasingly complemented by simulation, video analysis, three-dimensional anatomical models, structured assessment, and individualized feedback. The commentary by Yang on personalized video feedback for robotic partial nephrectomy training using realistic three-dimensional tumor-kidney models illustrates this transition. Rather than simply reproducing the operating room environment, contemporary training technologies seek to reproduce patient-specific anatomy and provide structured feedback regarding technical performance (Mourmouris et al.). This may represent one of the most important developments in robotic surgery. If surgical performance can be recorded, segmented, analyzed, and objectively evaluated, then training can progressively move from a largely experience-based model toward proficiency-based, data-informed surgical education.

## From robotic surgery to surgical intelligence

Taken together, the contributions to this Research Topic suggest that the next phase of robotic GU surgery will be defined by convergence. Robotic platforms will increasingly converge with artificial intelligence, computer vision, advanced imaging, three-dimensional reconstruction, surgical navigation, simulation, and large-scale surgical meta data. The objective should not be automation for its own sake. Instead, technology should augment the surgeon's ability to make better decisions, execute complex procedures more precisely, and provide patients with outcomes that matter to them. Artificial intelligence may ultimately assist in preoperative planning, identify anatomical structures, predict surgical difficulty, recognize procedural steps, provide real-time intraoperative guidance, and assess technical performance. However, the transition toward increasingly intelligent robotic surgery must be accompanied by rigorous validation, transparency, appropriate regulation, and careful consideration of patient safety and responsibility. The future is therefore unlikely to be defined by a simple transition from “robot-assisted” to “robot-performed” surgery. A more realistic and clinically meaningful trajectory is the emergence of human–robot collaboration, in which the surgeon retains responsibility for clinical judgment while increasingly sophisticated technological systems provide information, precision, prediction, and decision support.

## A glimpse into the future

The six contributions of this Research Topic demonstrate that robotic genitourinary surgery has already moved considerably beyond its original promise. Robotic technology is being used to refine functional outcomes, address increasingly complex renal tumors, perform sophisticated reconstruction, explore novel navigation systems, optimize postoperative pathways, and transform surgical education (Zeng et al.; Doganca et al.; Gu et al.; Jia et al.; Sun et al.; Mourmouris et al.; Yang).

Yet, these advances also expose the limitations of the current evidence base. Many innovations remain supported by retrospective or single-center experiences, relatively small cohorts, and heterogeneous definitions of outcomes. The next generation of research must therefore move beyond demonstrating technical feasibility. Comparative effectiveness, long-term functional and oncological outcomes, cost-effectiveness, external validation, reproducibility, and patient-reported outcomes will be essential. The ultimate measure of robotic evolution should not be the sophistication of the technology itself. It should be whether that technology allows us to deliver safer surgery, better cancer control, faster functional recovery, more precise reconstruction, more reproducible outcomes, and genuinely personalized care.

The journey from the promising past of robotic surgery to its even more exciting future has therefore only begun. The next major leap may not come from another robotic arm or another surgical approach, but from the integration of robotics with intelligence, imaging, data, and human expertise. The operating room of the future will not simply be robotic. It will be increasingly connected, adaptive, personalized, and intelligent.

